# Glaucoma and Psychotropics

**DOI:** 10.1192/j.eurpsy.2022.1207

**Published:** 2022-09-01

**Authors:** T. Cavaco, J. Rema, C. Rodrigues

**Affiliations:** Centro Hospitalar Universitário Lisboa Norte, Neurociências E Saúde Mental, Lisboa, Portugal

**Keywords:** glaucoma, psychotropic drugs

## Abstract

**Introduction:**

Glaucoma is a heterogeneous group of conditions which result in optic neuropathy and visual defects, majorly linked with the increase of intra-ocular pressure (IOP). It is known that psychotropic drugs have been implicated in drug induced angle-closure glaucoma, mostly through its anti-cholinergic effect.

**Objectives:**

Systematize the drugs most and least implicated in its appearance and worsening and understand the care needed on prescribing.

**Methods:**

A search on Pubmed database was made having in consideration the Mesh Terms Glaucoma and Psychotropic Drugs and its different classes. Specific searches were made when appropriate on different platforms.

**Results:**

Implications on the appearance and worsening of glaucoma are clear for tricyclic antidepressants. The evidence is not clear for SSRIs, SNRIs and mirtazapine, but they might be related with increased IOP. Other classes of antidepressants seem to be of lower risk. Antipsychotics do not seem to be greatly associated with angle closure, although there are some case reports. There are descriptions of the potencial use of haloperidol, anti-convulsive mood stabilizers, with exception of topiramate, melatonin and anti-dementia drugs on the treatment of this condition. In practice, benzodiazepines do not seem to precipate angle-closure. Methamphetamines are contraindicated. Eletroconvulsive therapy its an option.

**Conclusions:**

Although not prevalent, angle-closure glaucoma can have serious implications and culminate in irreversible blindness. In patients with known risk-factors its important to have it on consideration at the time of the prescription and warn on seeking immediate help if having acute ocular pain, redness and/ or cloudy vision.

**Disclosure:**

No significant relationships.

